# HbA1c below 7 % as the goal of glucose control fails to maximize the cardiovascular benefits: a meta-analysis

**DOI:** 10.1186/s12933-015-0285-1

**Published:** 2015-09-22

**Authors:** Pin Wang, Rong Huang, Sen Lu, Wenqing Xia, Haixia Sun, Jie Sun, Rongrong Cai, Shaohua Wang

**Affiliations:** Department of Endocrinology, Affiliated ZhongDa Hospital of Southeast University, No. 87 DingJiaQiao Road, Nanjing, 210009 People’s Republic of China; Department of Endocrinology, Sichuan Academy of Medical Science and Sichuan Provincial People’s Hospital, East District, No. 32, Section 2, 1st Ring Road (West), Chengdu, 610072 Sichuan People’s Republic of China; Department of Intensive Care Unit, Sichuan Academy of Medical Science and Sichuan Provincial People’s Hospital, No. 32, Section 2, 1st Ring Road (West), Chengdu, 610072 Sichuan People’s Republic of China

**Keywords:** Glucose control, Cardiovascular outcomes, HbA1c, Diabetes mellitus

## Abstract

**Objective:**

Whether lowering glycosylated haemoglobin (HbA1c) level below 7.0 % improves macro-vascular outcomes in diabetes remains unclear. Here, we aimed to assess the effect of relatively tight glucose control resulting in a follow-up HbA1c level of less or more than 7.0 % on cardiovascular outcomes in diabetic patients.

**Research design and methods:**

We systematically searched Medline, Web of science and Cochrane Library for prospective randomized controlled trials published between Jan 1, 1996 and July 1, 2015 that recorded cardiovascular outcome trials of glucose-lowering drugs or strategies in patients with type 2 diabetes mellitus.

**Results:**

Data from 15 studies involving 88,266 diabetic patients with 4142 events of non-fatal myocardial infarction, 6997 of major cardiovascular events, 3517 of heart failure, 6849 of all-cause mortality, 2084 of non-fatal stroke, 3816 of cardiovascular death were included. A 7 % reduction of major cardiovascular events was observed only when relatively tight glucose control resulted in a follow-up HbA1c level above 7.0 % (OR 0.93, 95 % CI 0.88–0.98; I^2^ = 33 %), however, the patients can benefit from reduction incidence of non-fatal myocardial infarction only when the follow-up HbA1c value below 7.0 % (OR 0.85, 95 % CI 0.74–0.96). Apart from the HbA1c value above 7.0 % (OR 1.22, 95 % CI 1.06–1.40), the application of thiazolidinediones (OR 1.39, 95 % CI 1.14–1.69) also increased the risk of heart failure, while the gliptins shows neutral effects to heart failure (OR 1.14, 95 % CI 0.97–1.34).

**Conclusions:**

Relatively tight glucose control has some cardiovascular benefits. HbA1c below 7.0 % as the goal to maximize the cardiovascular benefits remains suspended.

## Background

Diabetes is a chronic disease, and its rapid emergence worldwide has led to its classification as an epidemic. The life expectancy of an individual who is diagnosed with type 2 diabetes at 40 years of age is estimated to be shortened by approximately 6–7 years [1]. Coronary artery disease accounts for 75 % of deaths in patients with diabetes mellitus [2–4]. Glycosylated haemoglobin (HbA1c) level, the most commonly used indicator of blood glucose level, is closely associated with cardiovascular events and death [5]. A 1 % point increase in HbA1c level in diabetic patients generates an 18 % increased risk of cardiovascular events and a 12–14 % increase in mortality [5].

Although many factors were involved in diabetic complications such as age, gender, systolic blood pressure, and so on [6], intensive glucose control has been shown to reduce microvascular complications, such as retinopathy and nephropathy by UKPDS study [7], the degree to which it can reduce cardiovascular outcomes have been equivocal [8–10]. In ACCORD trial, a target HbA1c level of below 6.0 % assigned to a group subjected to intensive therapy, and the trial was terminated early, after a median of 3.5 years, because of a higher observed mortality rate among participants assigned to the intensive therapy group [9]. Despite inconsistent results of previous studies, a meta-analysis consisting of five randomized controlled clinical studies, UKPDS, PROactive, ADVANCE, VADT and ACCORD, showed that intensive glycaemic control reduced the odds ratio of non-fatal myocardial infarction by 17 % without increasing mortality rate [11]. The American Diabetes Association recommends lowering the HbA1c level below approximately 7.0 % to reduce microvascular complications in many non-pregnant adults [12]. However, reducing HbA1c levels to below 7.0 % reduces macro-vascular complications and mortality is still unclear. An investigation of diabetes mellitus by the Veterans Health Administration reported that half of the included 205,857 patients who received insulin and/or sulfonylureas had HbA1c levels of less than 7.0 %, and these individuals were found to be at high risk of adverse outcomes [13]. Because determining a target HbA1c value is just a preliminary expectation, the final results of same target glycemic control vary widely due to the complexity of clinical practice. The current meta-analysis assessed the effects of relatively tight glucose control resulting in a follow-up HbA1c level of below 7.0 % on a variety of cardiovascular outcomes.

## Methods

### Literature search strategy

We searched Medline, Web of Science and Cochrane Library for reports published in English between Jan 1, 1996 and July 1, 2015 using the following search terms: “diabetes mellitus” in combination with the terms “cardiovascular”, “macrovascular”, “complication”, and “glucose control”. We restricted the search to “Human species” and “randomized controlled trials”. A total of 6146 reports were further screened for inclusion by reviewing their titles, abstracts, or full texts. We also examined the reference lists of the identified articles previous meta-analyses to supplement the electronic search.

### Study selection

**Fig. 1:**
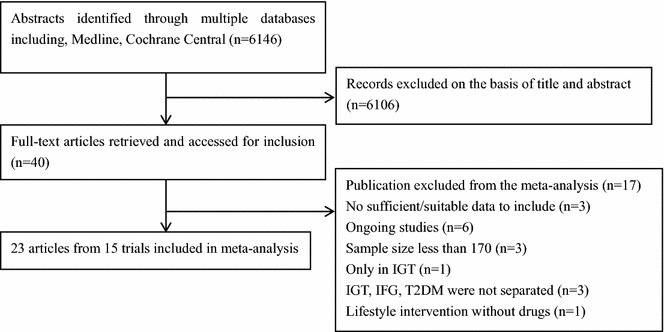
Study flow chart

Two independent researchers accessed the articles based on the following inclusion criteria: (1) randomized controlled clinical trials that compared cardiovascular risk of intensive lowering of glucose to a standard treatment regimen in type 2 diabetes mellitus and (2) trials performed on 1000 or more individuals with a minimum mean follow-up period of 1 year. Any disagreements were resolved by a third party or by consulting with experts. Twenty-three articles from 15 trials that met the inclusion criteria were included in this study (Fig. 1).

Seventeen trials were excluded for the following reasons: The DQDPS investigated patients with impaired glucose tolerance, and Leiter’s study reported outcomes, such as glucose level and weight, but did not assess cardiovascular outcomes [14, 15]. The ADOPT study mainly evaluated the effectiveness of rosiglitazone on indicators of glucose metabolism and did not assess cardiovascular outcomes [16]. The ORIGN, DREAM and UGDP trials assessed outcomes in patients with impaired fasting glucose, impaired glucose tolerance, and type 2 diabetes without separating them [17–19]. In the NAVIGATOR trial, pre-diabetes mellitus patients were treated with two drugs: valsartan and/or nateglinide [20]. The Steno-2 study, Kumamoto Study and Veterans Affairs study included a total of 160, 100 and 153 diabetic patients respectively, and none of those studies could provide sufficient evidence regarding the effects of glucose control [21–23]. The LOOK-AHEAD study investigated controlling glucose level with intensive lifestyle changes but did not record drug usage [24]. The other 6 studies that were excluded were still ongoing at the time of this meta-analysis and did not have sufficient data for inclusion [25–30].

### Data extraction

Three authors (PW, RH and SL) independently extracted information using standard data extraction forms as described in the Cochrane Handbook of Systematic Reviews of Interventions [31]. The extracted data included baseline demographic characteristics, such as age, diabetic duration, population, BMI, and HbA1c level (shown in Table 1 in "Appendix"), as well as outcomes, including non-fatal myocardial infarction, major cardiovascular events (cardiovascular death, non-fatal MI, and non-fatal stroke), all-cause mortality, cardiovascular death, non-fatal stroke, and heart failure. Disagreements were resolved by discussion with Professor SW.

**Table 1:** Baseline characteristics of included trials

Trial	UKPDS33	UKPDS34	PROactive	ADVANCE	ACCORD	HEART2D	VADT	RECORD	BARI2D	ADDITION	SAVOR-TIMI53	EXAMINE	DIGAMI1	AleCardio	TECOS
Intervention	Intensive policy with a sulphonylura or insulin vs conventional policy with diet	intensive blood-glucose control policy with metformin vs diet alone	Addition of pioglitazone or placebo to usual diabetes therapy	Intensive (glicazide plus other drugs) vs standard glucose control	Intensive therapy vs standard therapy	Prandial vs basal strategy	Intensive or standard glucose control	Addition of rosiglitazone or combination of metformin and sulfonylurea	Insulin sensitization vs insulin provision therapy	Routine vs intensive treatment of multiple risk factors	Addition of Saxagliptin vs placebo to usual diabetes therapy	Addition of Alogliptin vs placebo to usual diabetes therapy	Intensified insulin-based glycaemic control vs conventional glucose-lowering treatment	Aleglitazar 150 μg or placebo daily	Add sitagliptin or placebo to existing therapy
Publication year	1998	1998	2005	2008	2008	2009	2009	2009	2009	2011	2013	2013	2014	2015	2015
Location	23 centers in England	15 centers in England	321 centers in 19 countries^a^	215 centers in 20 countries^b^	77 centers in USA	105 centers in 17 countries^c^	20 centers in USA	364 centers in 25 countries^d^	49 centers in 6 countries^e^	334 practices in Denmark, Netherlands, and UK	788 sites in 26 countries^f^	898 sites in 48 countries^g^	19 hospitals in Swedish	720 hospitals in 26 countries^h^	673 sites in 38 countries^i^
Study design	Randomized, Open label	Randomized, Open label	Randomized, Placebo-controlled	Factoria randomized trial	Randomized, 2 × 2 factorial design	Randomized, open label	Open label, permuted-block design	Randomized, open label	Randomized, 2by2 factorial design	Cluster-randomized, parallel-group trial	Randomized, double-blind, placebo-controlled	Randomized, double-blind	Randomized, open-label	Randomized, double blind, placebo controlled trial	Randomized, double blind, placebo controlled, event driven trial
Number of patients	3867	1704	5238	11,140	10,251	1115	1791	4447	2368	3055	16,492	5380	1240	7226	14,671
Duration of diabetes	Newly diagnosed	Newly diagnosed	8 ± 6	8 ± 6^†^	10	9 ± 7.2^†^	11.5 ± 7.5^†^	7 ± 4.8^†^	10.4 ± 8.7	Screened diabetes	10.3 ± 2.8^†^	7.2 ± 2.8^†^	10.5 ± 5.4^†^	8.6 ± 7.7	9.4 ± 2.6*
Population	Newly diagnosed T2DM	Newly diagnosed T2DM	T2DM with macrovascular disease	T2DM, history of macrovascular or microvascular disease or at least one other CV risk factor	T2DM with established CVD or additional CV risk factors	T2DM after acute MI	T2DM	T2DM	T2DM and heart disease	Screen detected T2DM	T2DM with history of CV event or at risk for	T2DM with ACS within 15–90 before randomization	T2DM and acute MI	Type 2 diabetes with hospitalized for ACS	Type 2 diabetes with established cardiovascular disease
Average follow up (years)	10.1	10.7	2.9	5.0	3.5	2.6	5.6	5.5	5.3	5.3	2.1	18 months	3.4	104 weeks	3
Age	54 (48–60)*	53 ± 8.6^†^	62 ± 8	66 ± 6	62 ± 7	61 ± 9.7	60 ± 9	58 ± 8	62 ± 9	60 ± 6.8	65 ± 8.5	61	67.5 ± 9.4	61 ± 10	66.0 ± 8.0
BMI (kg/m^2^)	27.5 ± 5.2	31.7 ± 4.9^†^	31 ± 5	28 ± 5	32 ± 6	29.1 ± 4.8^†^	31 ± 4	31.5 ± 4.7^†^	31.7 ± 6.0	31.6 ± 5.6	31.1 ± 5.6	28.7 ± 11.6	27.1 ± 4.3	28.6 ± 1.7	30.2 ± 5.7
HbA1c at baseline (100 %)	7.1 ± 1.5	7.2 ± 1.5†	7.9 ± 1.4	7.5 ± 1.6	8.3 ± 1.1	8.3 ± 1.5	9.4 ± 2.0	7.9 ± 0.7	7.7 ± 1.6	7.0 ± 1.6	8.0 ± 1.4	8.0 ± 1.1	8.0 ± 1.9	7.8 ± 1.7^†^	7.3 ± 0.7
Intervention group	7.0 ± 1.5*	8.3	7.0	6.5 ± 0.99	6.4 ± 0.6	7.7 ± 0.1	6.9 ± 0.6*	7.5	7.0 ± 1.2	6.6 ± 0.95	7.7	7.7	7.3 ± 1.9	7.03	7.1
Conventional group	7.9 ± 1.4*	8.8	7.6	7.2 ± 1.4	7.5 ± 0.7	7.8 ± 0.1	8.4 ± 1.1*	7.8	7.5 ± 1.4	6.7 ± 0.95	7.9	8.0	7.6	7.77	7.5
Change in HbA1c^‡^	0.1	−1.1	0.9	1.0	1.9	0.6	1.5	0.4	0.7	0.4	0.3	0.3	0.7	0.8	0.2

Data are presented as mean ± SD, or median (interquartile range), unless otherwise specified

*T2DM* type 2 diabetes mellitus, *BMI* body mass index, *HbA1c* glycosylated hemoglobin

^a^Austria, Belgium, Denmark, Estonia, Finland, Czeh Repulic, France, Germany, Hungary, Italy, Latvia, Lithuania, Netherlands, Norway, Poland, Siovakia, Sweden, Switzerland and UK

^b^Austria, Canada, China, Czeh Repulic, Estonia, France, Germany, Hungary, India, Ireland, Italy, Lithuania, Malaysia, Netherlands, New Zealand, Philippines, Poland, Russia, Slovakia, and UK

^c^Canada, Croatia, Czech Republic, Germany, Hungary, India, Israel, Lebanon, Poland, Romania, Russian Federation, Slovakia, Slovenia, South Africa, Spain, Turkey, and UK

^d^Australia, Belgium, Bulgaria, Croatia, Czech Republic, Denmark, Estonia, Finland, France, Germany, Greece, Hungary, Italy, Latvia, Lithuania, Netherlands, New Zealand, Poland, Romania, Russia, Slovakia, Spain, Sweden, Ukraine, and UK

^e^A, Canada, Brazil, Mexico, the Czech Republic and Austria

^f^Argentina, Australia, Brazil, Canada, Chile, China, Czech Republic, France, Germany, Hong Kong, Hungary, India, Israel, Italy, Mexico, Netherlands, Peru, Poland, Russian Federation, South Africa, Spain, Sweden, Taiwan, Thailand, UK and USA

^g^Argentina, Australia, Belgium, Brazil, Bulgaria, Canada, Chile, Colombia, Croatia, Czech Republic, Denmark, Egypt, Finland, France, Germany, Great Britain, Greece, Guatemala, Hong Kong, Hungary, India, Israel, Italy, Japan, Korea, Kuwait, Latvia, Lithuania, Malaysia, Mexico, New Zealand, Peru, Philippines, Poland, Portugal, Puerto Rico, Romania, Russia, Serbia, Slovakia, South Africa, Spain, Sweden, Taiwan, Thailand, Ukraine, United Arab Emirates and USA

^h^Argentina, Australia, Brazil, Canada, China, Czech Republic, France, Germany, Hungary, India, Ireland, Italy, Korea, Malaysia, Mexico, The Netherlands, New Zealand, Poland, Romania, Russia, Spain, Sweden, Thailand, United Kingdom, United States

^i^Argentina, Australia, Belgium, Bulgaria, Brazil, Canada, Chile, China, Colombia, Czech Republic, Estonia, Finland, France, Germany, Great Britain, Hong Kong, Hungary, India, Israel, Italy, Lithuania, Latvia, Malaysia, Netherlands, Norway, New Zealand, Poland, Romania, Russia, Singapore, Slovakia, South Africa, South Korea, Spain, Sweden, Taiwan, Turkey, Ukraine, United States

* The SD value were estimated from IQR according to Cochrane handbook

^†^Combined data by sample size according to Cochrane handbook

^‡^Calculated by baseline HbA1c level and HbA1c level in intervention group

### Statistical analysis

Data from the 15 trials included in this meta-analysis were stratified according to whether patients had follow-up HbA1c levels of <7.0 or ≥7.0 %. Odds ratios and 95 % CIs were calculated from dichotomous frequency data allocated from each trial. The I^2^ statistic was used to quantify statistical heterogeneity between trials [32]. All analyses were performed with a fixed-effects model when I^2^ <50 % and a randomized-effect model when I^2^≥ 50 % using Review Manager 5.0. The probability of publication bias was assessed by funnel plots and the Egger test [33]. Meta-regression analyses were used to identify the risk factors of heart failure between trials with Stata version 11.0 software. All p values are two-sided; p <0.05 was considered statistically significant.

## Results

A total of 88,266 patients were included in this meta-analysis: 45065 were randomized to relatively tight glucose control group, and 43210 were randomized to conventional therapy. The general baseline characteristics of the participants are shown in Table 1 in "Appendix". The mean participant age was 63 years. Among the included studies, UKPDS33, UKPDS34 and ADDITION enrolled newly diagnosed or screened diabetes patients, while the participants in the other studies had a mean diabetic duration of 8 years. The mean follow-up period ranged from 18 months to 10 years. The follow-up period of DIGAMI1 ranged from 0 to 21.8 years to observe the effect of glucose control on mortality in older patients who experienced myocardial infarction (mean age = 68 years). Ten studies enrolled diabetic patients with a history of macrovascular disease [9, 10, 34–41], and HEART2D, EXAMIN, and DIGAMI1 only enrolled patients who had recently experienced adverse coronary events. Most patients were overweight, with a mean BMI of 30 kg/m^2^. The baseline HbA1c level was 7.8 %, and the final HbA1C levels in the intensive glucose control and conventional groups were 7.1 and 7.6 %, respectively. The main interventions for the relatively tight glucose control group and the conventional group are shown in Table 1 in "Appendix".

### Outcomes of relatively tight glucose control stratified by follow-up HbA1c level

**Fig. 2:**
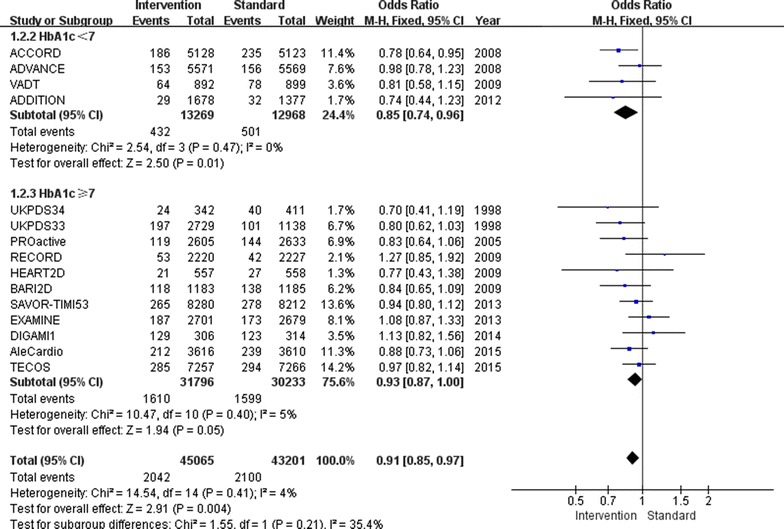
Risk of non-fatal myocardial infarction stratified by HbA1c level of 7.0 %

**Fig. 3:**
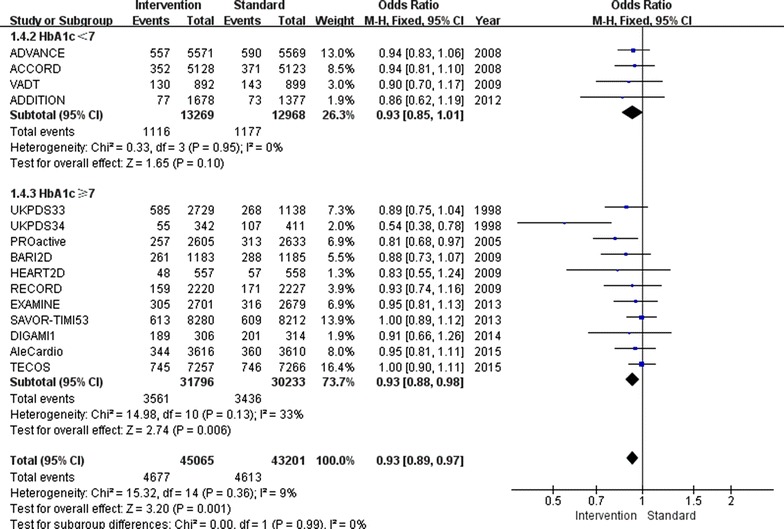
Risk of major cardiovascular events stratified by HbA1c level of 7.0 %

Overall, relatively tight glucose control decreased the incidence of non-fatal myocardial infarction by 9 % (OR 0.91, 95 % CI 0.85–0.97; I^2^ = 4 %; Fig. 2) and the incidence of major cardiovascular events by 7 % (OR 0.93, 95 % CI 0.89–0.97; I^2^ = 9 %; Fig. 3). Furthermore, major cardiovascular events were decreased by 7 % (OR 0.93, 95 % CI 0.88–0.98; I^2^ = 33 %) when the follow-up HbA1c level was higher than 7.0 %, however, only when the follow-up HbA1c level was lower than 7.0 %, the benefit of relatively tight glucose control in regards to the prevention of non-fatal myocardial infarction was gained (OR 0.85, 95 % CI 0.74–0.96; I^2^ = 0 %).

**Fig. 4:**
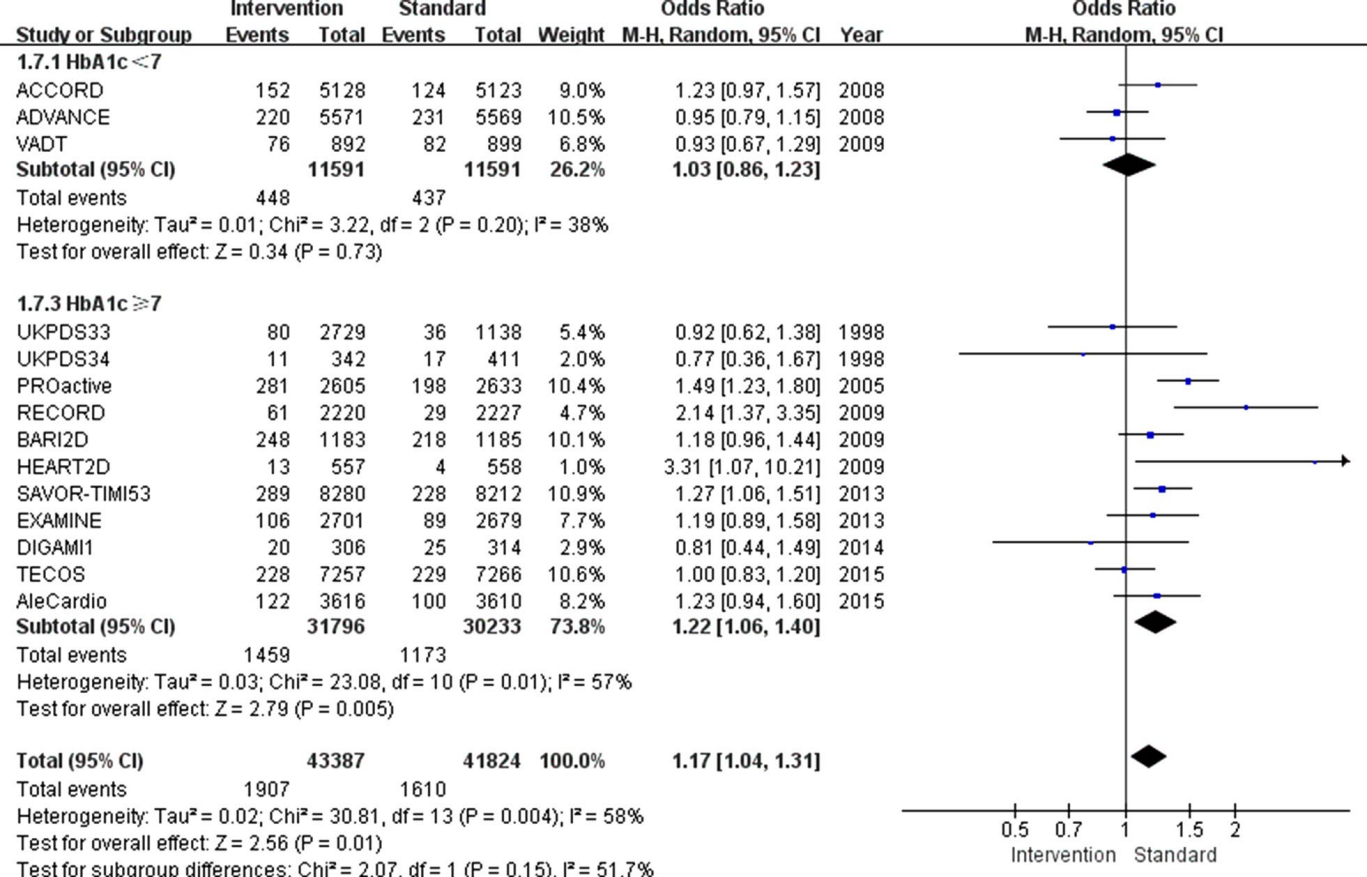
Risk of heart failure stratified by HbA1c level of 7.0 %

There was also a 17 % increase in the incidence of heart failure (OR 1.17, 95 % CI 1.04–1.31; I^2^ = 58 %; Fig. 4) in the relatively tight glucose control group compared to conventional group. The subgroup with a follow-up HbA1c level above 7.0 % showed an increased incidence of heart failure of 22 % (OR 1.22, 95 % CI 1.06–1.40; I^2^ = 57 %), while the subgroup with a follow-up HbA1c level below 7.0 % showed no increase in the incidence of heart failure (OR 1.03, 95 % CI 0.86–1.23; I^2^ = 38 %).

**Fig. 5:**
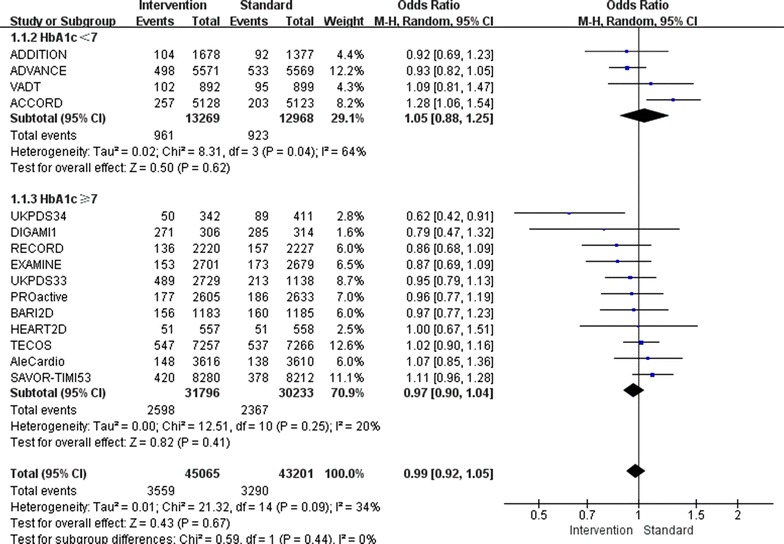
Risk of all-cause mortality stratified by HbA1c level of 7.0 %

**Fig. 6:**
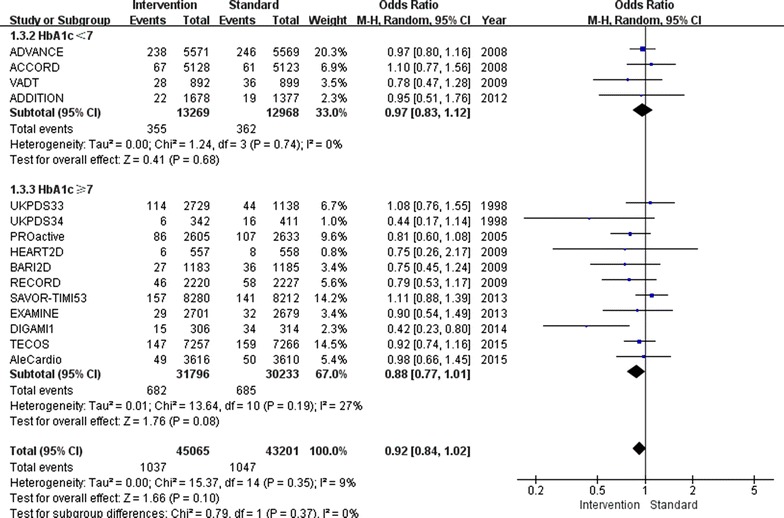
Risk of stroke stratified by HbA1c level of 7.0 %

**Fig. 7:**
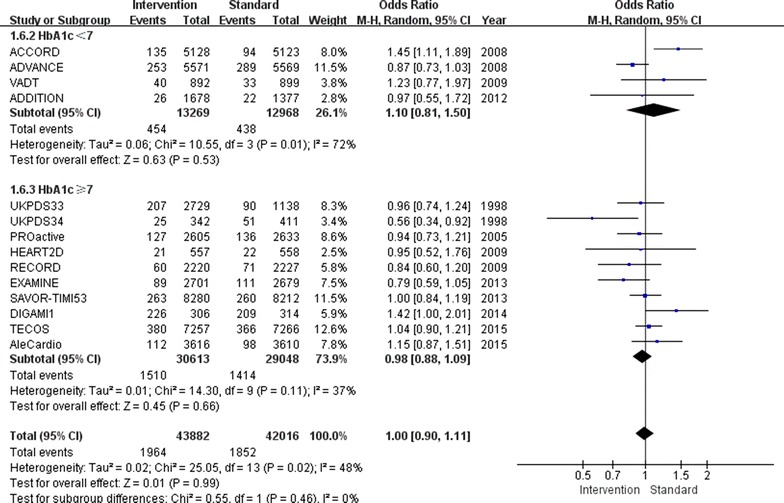
Risk of cardiovascular death stratified by HbA1c of 7.0 %

Regardless of whether the follow-up HbA1c level was below or above 7.0 %, no differences between the relatively tight glucose control and conventional groups were found for all-cause mortality (OR 0.97, 95 % CI 0.90–1.04; I^2^ = 20 %; Fig. 5), non-fatal stroke (OR 0.92, 95 % CI 0.84–1.02; I^2^ = 9 %; Fig. 6) or cardiovascular death (OR 1.00, 95 % CI 0.90–1.11; I^2^ = 48 %; Fig. 7).

The funnel plot and Egger test results showed no underlying publication bias.

### Meta-regression analysis and stratification according to relevant factors

In an attempt to determine other sources of surplus nuances among the trials, meta-regression analyses of the glucose-lowering strategies for the relatively tight glucose control group, history of cardiovascular disease, follow-up period, BMI, age and diabetic duration were performed. Among these variables, the correlation between non-fatal myocardial infarction and relatively tight glucose control was stronger in patients with a BMI higher than 30 kg/m^2^ (OR 0.89, 95 % CI 0.82–0.96; I^2^ = 1.4 %; Table 2 in "Appendix"). In addition to a follow-up HbA1c level above 7 %, the application of thiazolidinediones (TZDs) (OR 1.39, 95 % CI 1.14–1.69, I^2^ = 59.2 %) increased the risk of heart failure, while the dipeptidyl peptidase inhibitors (gliptins) shows neutral effects to heart failure (OR 1.14, 95 % CI 0.97–1.34, I^2^ = 41.9; Table 3 in "Appendix").

**Table 2:** The pooled odds ratio of myocardial infarction stratified by BMI

	Myocardial infarction			
	Intervention	Conventional	Odds ratio (95 %CI)	I^2^ (%)
Overall	2042/45,065	2100/43,192	0.91 (0.85, 0.97)	3.9
BMI				
<30 kg/m^2^ (UKPDS33, ADVANCE, HEART2D, EXAMINE, DIGAMI1, AleCardio)	899/15,480	819/13,859	0.95 (0.85, 1.05)	10.1
≥30 kg/m^2^	1143/29,585	1281/29,333	0.89 (0.82, 0.96)	1.4

**Table 3:** The pooled odds ratio of heart failure stratified by different glucose lowering strategies

	Heart failure			
	Intervention	Conventional	Odds ratio (95 %CI)	I^2^ (%)
Overall	1907/43,387	1610/41,824	1.17 (1.04, 1.31)	57.8
Glucose-lowering strategies				
Intensive control (ACCORD, ADVANCE, VADT, DIGAMI1, UKPDS34,33)	559/14,968	515/13,454	1.00 (0.88, 1.13)	0.0
Thiazolidinediones (PROactive, RECORD, BARI2D, AleCardio)	712/9624	545/9655	1.39 (1.14, 1.69)	59.2
Dipeptidyl peptidase inhibitors (SAVOR-TIMI53, EXAMINE, TECOS)	623/18,238	546/18,157	1.14 (0.97, 1.34)	41.9
Prandial vs basal strategy (HEART2D)	13/557	4/558	3.31 (1.07, 10.21)	

**Fig. 8:**
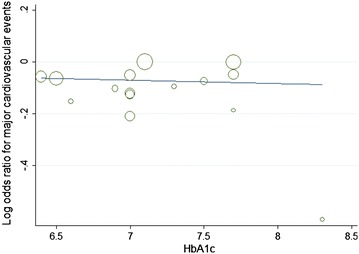
Odds ratio of major cardiovascular events in relation to follow-up HbA1c level

**Fig. 9:**
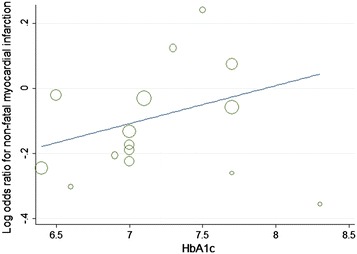
Odds ratio of myocardial infarction in relation to follow-up HbA1c level

**Fig. 10:**
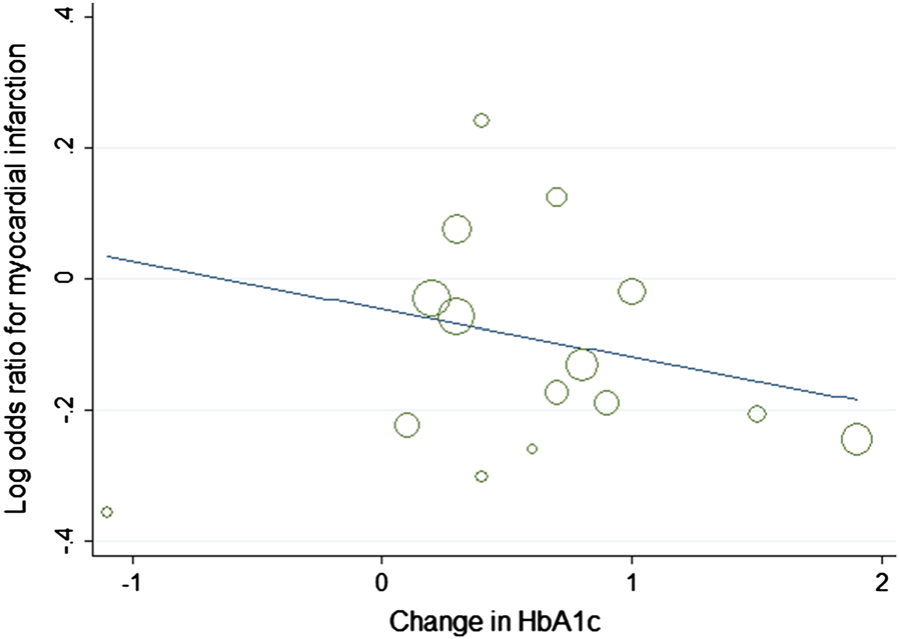
Odds ratio of myocardial infarction in relation to HbA1c change

**Fig. 11:**
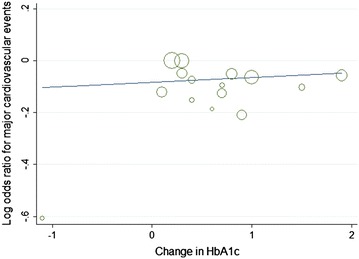
Odds ratio of major cardiovascular events in relation to HbA1c change

Furthermore, with each 1 % decrease in HbA1c level between trials associated with a marginal 2 % increase in major cardiovascular events (OR 0.98, 95 % CI 0.82–1.05; Fig. 8), without decrease in non-fatal myocardial infarction (OR 1.12, 95 % CI 0.96–1.31; Fig. 9).The mean HbA1c change of HbA1c below 7 % subgroup was 1.3 %, and in the HbA1c above 7 % subgroup was 0.3 %, with each 1 % increase in HbA1c change associated with marginal 7 % decrease in non-fatal myocardial infarction (OR 0.93, 95 % CI 0.82–1.05; Fig. 10), limited association were found between HbA1c change and major cardiovascular events (OR 1.02, 95 % CI 0.93–1.13; Fig. 11).

## Discussion

The results of the current meta-analysis were consistent with previous studies and showed that relatively tight glucose control in type 2 diabetes mellitus patients has cardiovascular benefits, namely reducing the incidences of non-fatal myocardial infarction and major cardiovascular events without increasing all-cause mortality. Interestingly, when the follow-up HbA1c level was above 7.0 %, the incidence of major cardiovascular events was obviously decreased, but the benefits in regard to the prevention of non-fatal myocardial infarction only can be obtained when the follow-up HbA1c level was below 7.0 %. Each 1 % decrease in HbA1c level are associated with a marginal 2 % increase in major cardiovascular events, and each marginal 7 % decrease in non-fatal myocardial infarction are at a cost of 1 % increase in HbA1c change. In spite of the HbA1c level, the increased risk of heart failure was closely associated with the application of thiazolidinediones, not gliptins.

Though the ACCORD [9] trial was stopped because of the high incidence of cardiovascular outcomes, but the latter ADVANCE [10] trial and meta-analysis suggested tight glucose control can lead to an obvious cardiovascular benefits especially non-fatal myocardial infarction [11]. And about half of the patients who were receiving hypoglycemic therapy had a HbA1c level of less than 7.0 % in the recent investigation [13]. Our study elaborate the effects of glycemic control on cardiovascular outcomes from the results of glycemic control which is the follow-up of HbA1c, and our study found that the incidence of major cardiovascular events, including non-fatal stroke, non-fatal myocardial infarction and cardiovascular death were not decreased in the patients of HbA1c controlled below 7 % compared to the group of HbA1c above 7 %, though the incidence of non-fatal myocardial infarction was reduced when the HbA1c level were controlled below 7 %. Based on the above findings, we inferred that strict glycaemic control targeting a follow-up HbA1c level below 7.0 % may increase the risk of non-fatal stroke and cardiovascular death; however, a separate analysis in the current study displayed no increased risk of non-fatal stroke or cardiovascular death. To obtain better blood glucose control, additional glucose lowering drugs must inevitably be used, thus, leading to redundant weight gain and severe hypoglycaemia, which both increased the risk of acute diabetic complications and likely offset the reduced incidence of myocardial infarction that follows intensive therapy [42]. Additionally, our analysis also showed that to obtain non-fatal myocardial infarction benefits by glucose control are at a great cost of the HbA1c level change. In our clinical practice, the ultimate goal of strict glycaemic control is to reduce diabetic complications and the incidence of fatal events and to increase the patient survival rate, not just once or twice reduced incidence of nonfatal myocardial infarction [43]. Taken together, a follow-up HbA1c level of 7.0 % is the critical control point for intensive therapy; our study suggests that controlling HbA1c below 7.0 % could not maximize the cardiovascular benefits, and the disadvantages outweigh the advantages.

Although there was some heterogeneity across the included studies, the results of this meta-analysis indicate that glucose control increases the risk of heart failure, and the subgroup with a follow-up HbA1c above 7.0 % have a greater risk of heart failure. Previous studies have demonstrated that every percentage point increase in HbA1c level results in a 15 % increase in the risk of congestive heart failure [44]. Advanced glycation end-products, oxidative stress and altered myocardial metabolism are probably involved in systolic and diastolic dysfunction and eventually cause heart failure, especially among diabetic patients with a history of heart disease [45–48]. Additionally, hyperglycaemia induces insulin secretion, which can increase the preload of the heart and decrease cardiac output [49]. Furthermore, elevated levels of glucose and insulin in the blood can activate the sympathetic nervous system, which has been implicated in the development of heart failure [50]. In addition, our meta-regression analysis further showed that the strategies of intensive therapy are closely associated with heart failure, especially among patients taking PPAR agonists. PPAR agonists cause fluid retention and diastolic dysfunction in susceptible patients and result in haemodynamic consequences that can cause heart failure [51]. It is worth pointing out that not the same with the recent meta-analysis which showed that gliptins induce heart failure in diabetic patients and patients at risk of developing T2DM [52], our study shows the effects of gliptins on heart failure is neutral.

This study had the inherent limitations of any meta-analysis that results from the use of published data, including the absence of standardization in study design, duration of follow-up, strategy of intensive glycaemic control, characteristics of the study populations, and end-point definitions. Another limitation was the search strategies used, which could have generated publication bias, leading to a misinterpretation of the results. Fortunately, the trials included in this analysis were mostly large-scale clinical trials with low heterogeneity, which effectively avoided the inaccurate results that can be generated by studies with small sample sizes. Additionally, ADDITION did not record information related to heart failure, so our analysis of that variable was based on incomplete data. Another point need to be considered is the choice of the indicator of glucose control. The mean HbA1c level and HbA1c change were used in this study, but the marginal value of the results suggested mean HbA1c level may not be a sensitive predictor for cardiovascular complication of diabetes mellitus. Other HbA1c index such as HbA1c variability especially intra-individual mean (HbA1c-MEAN) or haemoglobin glycation index which showed a better association with cardiovascular risk in diabetes may be a better index of glucose control [53, 54]. Other factors such as blood pressure, blood lipid, inflammatory biomarkers like C-reactive protein (CRP), monocyte chemotactic protein-1 (MCP1) and asymmetric dimethyl arginine (ADMA) could also be involved in the development of diabetic complications and affect the interpretation of outcomes [55–57]. At the same time, recommendations regarding type 2 diabetic patients’ treatments have focused on personalizing HbA1c targets which could be a better solution for diabetes with cardiovascular complications [58]. Other interventions such as lifestyle change, intensive blood pressure or blood lipid control should be considered in diabetes mellitus [24, 59].

## Conclusion

This meta-analysis indicates that intensive glycaemic control has cardiovascular benefits and does not increase all-cause mortality. However, lowering the HbA1c level below 7.0 % does not appear to maximize the cardiovascular benefits, although the risk of non-fatal myocardial infarction was reduced. Further research is still necessary to explore the different treatment regimens of diabetes mellitus.

## Appendix

See Tables 1, 2, and 3.
